# Fruits Vinegar: Quality Characteristics, Phytochemistry, and Functionality

**DOI:** 10.3390/molecules27010222

**Published:** 2021-12-30

**Authors:** Driss Ousaaid, Hamza Mechchate, Hassan Laaroussi, Christophe Hano, Meryem Bakour, Asmae El Ghouizi, Raffaele Conte, Badiaa Lyoussi, Ilham El Arabi

**Affiliations:** 1 Laboratory of Natural Substances, Pharmacology, Environment, Modeling, Health, and Quality of Life, Faculty of Sciences Dhar El Mahraz, University Sidi Mohamed Ben Abdellah, Fez, P.O. Box 1796, Morocco; driss.ousaaid@usmba.ac.ma (D.O.); hassan.laaroussi@usmba.ac.ma (H.L.); meryem.bakour@usmba.ac.ma (M.B.); asmae.elghouizi@usmba.ac.ma (A.E.G.); lyoussi@gmail.com (B.L.); ilham.elarabi@gmail.com (I.E.A.); 2 Laboratory of Inorganic Chemistry, Department of Chemistry, University of Helsinki, P.O. Box 55, FI-00014 Helsinki, Finland; 3 Laboratoire de Biologie des Ligneux et des Grandes Cultures, INRAE USC1328, University of Orleans, CEDEX 2, 45067 Orléans, France; christophe.hano@univ-orleans.fr; 4 Research Institute on Terrestrial Ecosystems (IRET)—CNR, Via Pietro Castellino 111, 80131 Naples, Italy; Raffaele.conte86@tiscali.it

**Keywords:** fruits vinegar, bioactive compounds, quality characteristics, biological properties, vinegar production

## Abstract

The popularity of fruits vinegar (FsV) has been increased recently as a healthy drink wealthy in bioactive compounds that provide several beneficial properties. This review was designed in the frame of valorization of fruits vinegar as a by-product with high value added by providing overall information on its biochemical constituents and beneficial potencies. It contains a cocktail of bioactive ingredients including polyphenolic acids, organic acids, tetramethylperazine, and melanoidins. Acetic acid is the most abundant organic acid and chlorogenic acid is the major phenol in apple vinegar. The administration of fruits vinegar could prevent diabetes, hypercholesterolemia, oxidative stress, cancer, and boost immunity as well as provide a remarkable antioxidant ability. The production techniques influence the quality of vinegar, and consequently, its health benefits.

## 1. Introduction

Fruits vinegar (FsV) is a popular natural product with multiple use purposes. It is remarkably appreciated and included in many people’s daily diet [1,2,3]. Fruits fermentation produces a bio liquid that contains several functional molecules [4,5] such as organic acids [5,6], polyphenols [7,8], melanoidins [9,10], and tetramethylpyrazine [11,12]. The production technique used has an impact on the quality of vinegar, while the vinegar-making process plays an important role in the removal and/or formation of new components. The traditional technique promotes the development of aroma and flavor due to the slow process of production [4,13,14]. 

Polyphenols and organic acids, mainly acetic acid, plays an important role in the beneficial properties provided by fruits vinegar [15]. Previously, it is shown that the administration of apple vinegar curbs the installation of hyperglycemia and hyperlipidemia induced by hypercaloric fed enriched in d-glucose in male and female rats [16]. Clinical studies demonstrated that apple vinegar regulates gene expression via the Mitogrn-Activated Protein Kinase (MAPK) pathway [17,18,19], and control blood glucose and lipids levels [19]. Interestingly, it contains a wide range of functional substances that exert their effects in synergy [3,20,21]. This review aimed to itemize the chemical composition of FsV and its therapeutics application scientifically proved. 

## 2. Methodology

All references used in the current review were collected via search engines including Google Scholar, Scopus, Science Direct, Web of Science, and Pub-Med using the following keywords: Fruits vinegar, Beneficial properties and vinegar, effects of fruits vinegar. The articles (203 articles) were evaluated for relevance and their scientific importance, and 119 articles were used to prepare the present review. Other articles were excluded for the purpose treated that they didn’t fall in the scope of the present review. 

## 3. Quality Characteristics 

FsV quality depends on the procedure conditions and raw matter. Vinegar-making process techniques influence the organoleptic properties of final vinegar [4]. The quality characteristics of local products determination are very important to promote vinegar marketing. It is named as natural means; it is completely safe and healthy [3,22]. The quality analysis provides the composition of each product in wanted and unwanted ingredients that can potentially be toxic. The quality evaluation requires a panel of analysis including determination of components content and sensory analysis. The acidity is considered a key factor to determine the vinegar quality and its content is at least 5 or 6 degrees [23,24,25]. The alcohol content is set to the maximum at 0.5 degrees for fruits vinegar and 1% for alcohol vinegar [23,25]. The same values are required by the Codex Alimentarius Norm 79/19 [26].

The physicochemical properties of FsV in terms of pH, acidity, electrical conductivity, and °Brix values are summarized in Table 1. The pH of fruits vinegar ranged from 2.40 to 3.90 [27,28,29,30]. Electrical conductivity (EC) is an important parameter to evaluate the ability of vinegar to allow the passage of electrical current and its relation with the mineral content of the sample. The values of EC depend on the raw matter used to produce vinegar [27,30]. The acidity is an important criterion to examine the quality of vinegar. High values of acidity observed in apple vinegar made in Turkey may be caused by the hyperoxygenation that occurs during the second step of the production of vinegar [29]. The second criterion used to determine vinegar quality is the ethanol content, the values reported for different vinegars range from 0.01 to 2.53 degrees. High values of ethanol content were observed in date vinegar from Iraq [31]. 

## 4. Chemical Characteristics of FsV

FsV has been the subject of numerous research studies during the last decades. Recently, studies are being carried out to determine and identify the phenolic composition of vinegar (Table 2). The nature and quantity of bioactive compounds present in vinegar are closely linked to the raw matter used to produce vinegar, the technique selected to produce vinegar, and the nature of microorganisms involved in the fermentation process [9,38,39]. The active molecules naturally present or new generated not only confer organoleptic properties such as astringency, taste, and color parameters [40,41]. Bioactive ingredients of vinegar play also an important role in the prevention and treatment of different human ailments [3,9,22,42].

FsV is considered a useful product in traditional medicine for the management of different illnesses such as diabetes [3,16,22]. As previously described, FsV is an excellent source of multiple bioactive ingredients such as phenolic acids, minerals, organic acids, and tetramethylpyrazine [9,11,29,43]. Phytochemicals present in FsV have numerous pharmacological properties including antidiabetic, antihyperlipidemic, antimicrobial, and anticancer [4,9,44].

**Table 2:** Phytochemical profile of fruits vinegar.

Variety	Country	Method of Vinegarmaking	Methods	Bioactive Compounds Identified	References
Grape vinegar	Turkey	Artisanal and industrial	HPLC-DAD	Gallic acid (16.36–18.23 mg/L), catechin (13.76–27.50 mg/L), epicatechin (4.96–8.20 mg/L), caffeic acid (6.30–10.30 mg/L), chlorogenic acid (0.16–3.73 mg/L), syringic acid (0.33–0.70 mg/L), *p*-coumaric acid (0.23–0.56 mg/L), and ferulic acid (0.06–0.35 mg/L)	[4]
Grape vinegar		Industrial	HPLC-PDA	Gallic acid (6 ± 2 mg/100 mL) and *p*-hydroxybenzoic acid (0.90 ± 0.05 mg/100 mL)	[7]
Apple vinegar				Gallic acid (0.8 ± 0.04 mg/100 mL), *p*-hydroxybenzoic acid (0.2 ± 0.1 mg/100 mL), catechin (2.4 ± 0.1 mg/100 mL), syringic acid (0.12 ± 0.02 mg/100 mL), caffeic acid (0.40 ± 0.01 mg/100 mL), and *p*-coumaric acid (0.08 ± 0.01 mg/100 mL)	
Apple vinegar		Artisanal	HPLC-DAD	Gallic acid (61.24 ± 2.21 mg/L), chlorogenic acid (347.70 ± 31.94 mg/L), catechin (68.20 mg/L), and caffeic acid (17.21 ± 0.33 mg/L)	[45]
Pomegranate vinegar				Gallic acid (67.80 ± 2.88 mg/L), catechin (47 ± 1.10 mg/L), and caffeic acid (13.41 ± 0.60 mg/L)	
Aromatic vinegar *	China	Artisanal	HPLC	Gallic acid, *p*-hydroxybenzoic acid, vanillic acid, catechin, caffeic acid, chlorogenic acid, syringic acid, ethyl gallate, *p*-coumaric acid, ferulic acid, sinapic acid, and rutin.	[46]
Grape vinegar	Turkey	Industrial	LC-DAD-ESI-MS/MS	Gallic acid (7.45–21.84 mg/L), tyrosol (11.54–17.68 mg/L), protocatechuic acid (7.21–11.05 mg/L), caftaric acid (1.76–15.83 mg/L), cholorogenic acid (0.09–1.77 mg/L), coutaric acid (0–1.95 mg/L) caffeic acid (0.11–2.58 mg/L), ferulic acid (0.01–0.21 mg/L), fertaric acid (0.03–0.83 mg/L), vanilic acid (0–2.58 mg/L), *p*-coumaric acid (0.02–0.45 mg/L), syringic acid (1.24–9.04 mg/L), procyanidin B2 (0.09–3.11 mg/L), catechin (3.73–27.11 mg/L), epicatechin (0.57–15.13 mg/L), quercetin-3-*O*-galactoside (0.04–0.39 mg/L), kaempferol-3-*O*-rutinoside (0–0.04 mg/L), rutin (0.02–0.20 mg/L), isorhamnetin-3-*O*-glucoside (0.05–0.09 mg/L), and quercetin (0.06–0.69 mg/L).	[8]
Apple vinegar				Gallic acid (0.47–2.57 mg/L), protocatechuic acid (1.15–6.35 mg/L), cholorogenic acid (2.96–16.29 mg/L), caffeic acid (0.19–1.77 mg/L), vanilic acid (0.63–3.42 mg/L), *p*-coumaric acid (0.13–0.81 mg/L), procyanidin B2 (0.12–1.35 mg/L), catechin (0.14–0.95 mg/L), epicatechin (0.04–1.36 mg/L), luteolin-3-*O*-rutinoside (0.30–1.98 mg/L), isorhamnetin-3-*O*-rutinoside (0.10–0.63 mg/L), isorhamnetin-3-*O*-glucoside (0.08–0.48 mg/L), kaempferol-3-*O*-glucoside (0.03–0.20 mg/L), quercetin-3-*O*-rhamnoside (0.20–3.41 mg/L), quercetin (0.20–1.41 mg/L), rutin (0.04–0.29 mg/L), luteolin (0.27–1.63 mg/L), apigenin0.02–0.13 mg/L), phloretin (0.59–7.86 mg/L), and phloridzin (7.64–44.35 mg/L).	
Apple vinegar	Japan	Industrial	LC-MS	Chlorogenic acid (3.1–19.6 mg/100 mL), 4-*p*-coumaric acid (0–0.21 mg/100 mL), isomer of *p*-coumaroyquinic acid (0–1.3 mg/100 mL), 5-hydroxymethylfurfural (2.7–4.1 mg/100 mL), protocatechic acid (0–0.41 mg/100 mL), p-hydroxybenzoic acid (0–0.77 mg/100 mL), caffeic acid (0–0.76 mg/100 mL), isomer of chlorogenic acid (0–3.1 mg/100 mL), and *p*-coumaric acid (0–0.21 mg/100 mL)	[47]
Persimmon vinegar	China	Artisanal	HPLC	Gallic acid (22.91 ± 1.22 mg/L), (+/−)-catechin hydrate (0.16 ± 0.89 mg/L), chlorogenic acid (0.06 ± 0.12 mg/L), caffeic acid (0.04 ± 0.06 mg/L), *p*-coumaric acid (0.03 ± 0.21 mg/L), trans-ferulic acid (0.02 ± 0.11 mg/L), (-)-epicatechin gallate (0.13 ± 0.09 mg/L), and phloridzin (0.38 ± 0.12 mg/L)	[48]
Apple vinegar				Gallic acid (0.35 ± 0.02 mg/L), vanillic acid (0.06 ± 0.04 mg/L), chlorogenic acid (6.56 ± 0.43 mg/L), caffeic acid (3.03 ± 0.02 mg/L), *p*-coumaric acid (0.33 ± 0.28 mg/L), trans-ferulic acid (0.24 ± 0.07 mg/L), (-)-epicatechin gallate (0.77 ± 0.34), and phloridzin (1.76 ± 0.34 mg/L).	
Kiwifruit vinegar				Gallic acid (9.67 ± 0.59 mg/L), (+/−)-catechin hydrate (1.47 ± 0.34 mg/L), vanillic acid (1.77 ± 0.23 mg/L), chlorogenic acid (3.12 ± 0.21 mg/L), caffeic acid (0.04 ± 0.05 mg/L), *p*-coumaric acid (0.34 ± 0.01 mg/L), trans-ferulic acid (0.01 ± 0.03 mg/L), and phloridzin (0.49 ± 0.02 mg/L)	
Apple vinegar	Brazil	Industrial	HPLC-PDA	Phloretin-2′-β-d-glucoside (4.81–15.55 mg/L), 5-caffeoylquinic acid (20.62–26.85 mg/L), caffeic acid (0.51–3.87 mg/L), *p*-coumaric acid (1.16–2.03 mg/L), quercetin-3-rutinoside (2.69–4.65 mg/L), quercetin-3-d-galactoside (0.73–9.75 mg/L), quercetin-3-β-d-glucoside (1.58–3.45 mg/L), quercetin-3-d-xyloside (1.62–2.54 mg/L), quercetin-*O*-α-l-arabinofuranoside (0.85–1.34 mg/L), and quercetin-3-*O*-rhamnoside (1.13–3.37 mg/L).	[49]
Apple vinegar	China	Industrial	HPLC-PDA	Chlorogenic acid (0.11–10.91 µg/mL), protocatechuic acid (0.08–1.54 µg/mL), and *p*-coumaric acid (0.10–0.17 µg/mL	[5]
Red wine vinegar				Gallic acid (4.10–9.99 µg/mL), protocatechuic acid (0.47–1.38 µg/mL), *p*-coumaric acid (0.81–1.39 µg/mL), and caffeic acid (1.48–1.73 µg/mL)	
White wine vinegar				Protocatechuic acid (0.16–0.32 µg/mL), *p*-coumaric acid (0–0.18 µg/mL), caffeic acid (0–0.32 µg/mL), and ferulic acid (0–0.31 µg/mL)	
Balsamic vinegar				Gallic acid (7.50–12.56 µg/mL), protocatechuic acid (0–3.29 µg/mL), *p*-coumaric acid (1.17–1.97 µg/mL), and caffeic acid (0–3.58 µg/mL)	
Sour cherry vinegar	Turkey	Industrial	HPLC	Gallic acid (160–170 mg/mL), chlorogenic acid (45–55 mg/mL), *p*-coumaric acid (17–23 mg/mL), caffeic acid (3.5–4 mg/mL), ferulic acid (1.3–4.6 mg/mL), catechin (0.7–1 mg/mL), and epicatechin (1.7–3.5 mg/mL)	[50]
Palm vinegar	Thailand	Artisanal	LC-MS	Gallic acid (14.14 ± 0.07 µg/mL), catechin (8.61 ± 0.32 µg/mL), rutin (6.67 ± 0.03 µg/mL), isoquercetin (11.27 ± 0.12 µg/mL), and quercetin (10.33 ± 0.16 µg/mL)	[51]
Brow beer vinegar	Italy	Industrial	HPLC-DAD-ESI(+)-MS	Protocatechuic acid *O*-glucoside (7.42 ± 0.03 mg/L), 3-caffeoylquinic acid (40.01 ± 1.13 mg/L), (4-Hydroxyphenyl) acetic acid (11.84 ± 0.02 mg/L), 4-vinylguaiacol (10.22 ± 0.04 mg/L), Catechin 7 *O*-glucoside (8.84 ± 0.02 mg/L), 4-hydroxybenzoic acid (38.23 ± 0.05 mg/L), (3-hydroxyphenyl)acetic acid (18.95 ± 0.04 mg/L), catechin 5 *O*-glucoside (7.24 ± 0.06 mg/L), coumaric acid *O*-glucoside (4.90 ± 0.05 mg/L), cerulic acid *O*-glucoside (4.33 ± 0.02 mg/L), gallic acid (5.72 ± 0.04 mg/L), vanilic acid *O*-glucoside (10.25 ± 0.03 mg/L), gallocatechin (7.66 ± 0.10 mg/L), sinapic acid *O*-glucoside (14.03 ± 0.12 mg/L), catechin *O*-diglucoside (8.41 ± 0.04 mg/L), kaempferol *O*-glucoside (6.28 ± 0.04 mg/L), feruloylquinic acid (6.60 ± 0.15 mg/L), chlorogenic acid (18.30 ± 0.02 mg/L), (+)-catechin (7.89 ± 0.04 mg/L), (−)-epicatechin (7.78 ± 0.12 mg/L), caffeic acid (10.58 ± 0.08 mg/L), sinapic acid (15.5 ± 0.06 mg/L), apigenin *O*-glucoside (6.15 ± 0.02 mg/L), quercetin *O*-glucoside (7.05 ± 0.06 mg/L), cohumulone I (4.44 ± 0.02 mg/L), cohumulone II (6.58 ± 0.10 mg/L), 8-prenylnaringenin (2.33 ± 0.02 mg/L), 6-prenylnaringenin (1.86 ± 0.02 mg/L), humulone (5.62 ± 0.08 mg/L), and isohumulone (4.14 ± 0.03 mg/L)	[52]
Pineapple vinegar		Industrial	UHPLC-QTOF-MS	Catechol, peonidin, (+)-catechin 3-*O*-gallate, m-coumaric acid, 7,3’,4’-trihydroxyflavone, 4-vinylsyringol, ferulic acid, mullein, genistin, 3,4-dihydroxyphenylglycol, 4-ethylcatechol, 6-prenylnaringenin, gallic acid, kaempferol 3-*O*-xylosyl-glucoside, 6,8-Dihydroxykaempferol, spinacetin 3-*O*-glucosyl-(1-6)-[apiosyl(1-2)]-glucoside, and malvidin 3-*O*-arabinoside	[53]
Cherry vinegar	Spain	Industrial	UPLC-DAD	Gallic acid (2.08–2.99 mg/L), HMF (6.96–9.48 mg/L), protocatechuic acid (2.12–2.43 mg/L), caftaric acid (2.05–2.81 mg/L), furoic acid (2.46–16.53 mg/L), protocatechualdehyde (0.046–0.263 mg/L), *cis-p*-Coutaric acid (1.83–2.25 mg/L), *trans-p*-Coutaric acid (1.15–1.55 mg/L), tyrosol (24.6–28.9 mg/L), catequin (0.165–0.334 mg/L), caffeic acid (0.184–0.308 mg/L), vanillic acid (2.66–3.44 mg/L), syringic acid (2.16–5.44 mg/L), vanillin (1.05–2.97 mg/L), *cis*-*p*-coumaric acid (0.174–0.481 mg/L), syringaldehyde (0.50–5.12 mg/L), coniferyl aldehyde (0.959–2.85 mg/L), and sinapaldehyde (16.1–19.1 mg/L)	[54]
Sugarcane vinegar	China	Industrial	UPLC-MS	Benzoic acid (1.027 ± 0.07 mg/L), ferulic acid (1.1240.063 mg/L), quinic acid (0.031 ± 0.002 mg/L), chlorogenic acid (1.217 ± 0.063 mg/L), apigenin (0.004 ± 0 mg/L), kaempferol (0.003 ± 0.0001 mg/L), caffeic acid (0.005 ± 0.0001 mg/L), luteolin (0.005 ± 0.0001 mg/L), and *p*-coumaric acid (0.027 ± 0.0001 mg/L)	[55]
Citrus vinegar	Italy	Industrial	UPLC-UV	Gallic acid (2.62–5.63 mg/L), neochlorogenic acid (2.69–5.83 mg/L), chlorogenic acid (2.95–58.51 mg/L), vanillic acid (0.47–3.64 mg/L), caffeic acid (1.39–3.64 mg/L), epicatechin (0–2.91 mg/L), procyanidin (0–9.43 mg/L), rutin (1.76–146.3 mg/L), quercetin (0.23–8.62 mg/L), eriocitrin (0.27–13.20 mg/L), neoeriocitrin (53.41–513.30 mg/L), narirutin (3.05–18.24 mg/L), naringin (61.19–700.56 mg/L), hesperidin (12.15–92.12 mg/L), neohesperidin (63.51–366.93 mg/L), didymin (1.73–9.82 mg/L), and hesperetin (0–15.54 mg/L)	[37]

* Vinegar mixed with other aromatic plants.

### 4.1. Organic Acids 

Multiple organic acids were found in FsV including volatile organic acid (acetic acid, formic acid, propionic acid, butyric acid, and quinic acid) and non-volatile organic acids (lactic acid, malic acid, pyroglutamic acid, citric acid, and succinic acid) (Table 3) [29]. As a food-grade product, vinegar’s quality depends on its various characteristics, including aroma, which is the most important quality criterion [55]. Aroma is related to the presence of organic acids in FsV that are present naturally in raw matter or newly generated during the fermentation process. As stated in Table 2, acetic acid is the most abundant acid in vinegar with a proportion of about 92.64–93.22% [29,55], and followed by succinic acid with a percentage of 3.92 to 6.43%, while, oxalic acid and malic acid were present at lowest quantities [56]. The content of organic acids changed during the production process of vinegar, and it showed the lowest content of organic acids during alcoholic fermentation with a gradual increase which was maintained during the second phase (acetic fermentation) [46]. It proved that these bioactive compounds exhibit several health benefits [24,57,58,59,60,61,62]. Acetic acid as the main organic acid in vinegars is the key factor of numerous beneficial properties through the activation of the MAPK pathway, which induces reduction of blood glucose, increases glycogen storage, reduces triglycerides levels, increases insulin sensitivity, and decreases insulin resistance [18]. The aforementioned features revealed that acetic acid is the main acid of vinegar that can ameliorate metabolic disorders and ameliorate disease markers [16,18,22,63,64,65,66,67,68,69,70,71]. 

### 4.2. Mineral Content 

The mineral composition of FsV is related to the raw matter. It plays an important role of human nutrition. Minerals are involved in several physiological functions such as blood pressure regulation, muscles contraction, blood cells production, maintaining bones and teeth healthy, sleep modulator, and maintenance of nerve functioning [75,76,77]. Vinegars are the potential source of mineral elements including potassium, sodium, calcium, iron, zinc, magnesium, phosphorus, and copper [78,79]. Zhang et al. [80] reported concentrations of different minerals in wood vinegar as 7.66 ± 0.80 (K), 13 ± 0.78 (Ca), 1.98 ± 0.34 (Mg), 3751 ± 60 (Fe), 23.7 ± 0.43 (Mn), and 0.166 ± 0.16 (Zn) mg/kg. Another study also found 153.8 ± 5.22 and 131.2 ± 4.29 (K), 21.85 ± 1.502 and 15.37 ± 0.734 (Na), 30.04 ± 0.522 and 6.715 ± 0.2967 (Ca), and 10.90 ± 0.087 and 6.901 ± 0.1194 mg/g for grape vinegar and apple vinegar respectively [81]. Various reports evoked the concentration of minerals present in vinegars are presented in Table 4. 

## 5. Beneficial Properties of FsV

The double fermentation of fruits furnishes a healthy product that contains an amount of biologically active components. Literature data reported that fruits vinegar consumption is positively counteracting against numerous diseases [3,32,39,42,62,67,84,85,86]. 

### 5.1. Antihyperglycemic Effect 

The beneficial antihyperglycemic effects of FsV are partitioned as follows: 

#### 5.1.1. Animal Studies 

**Figure 1:**
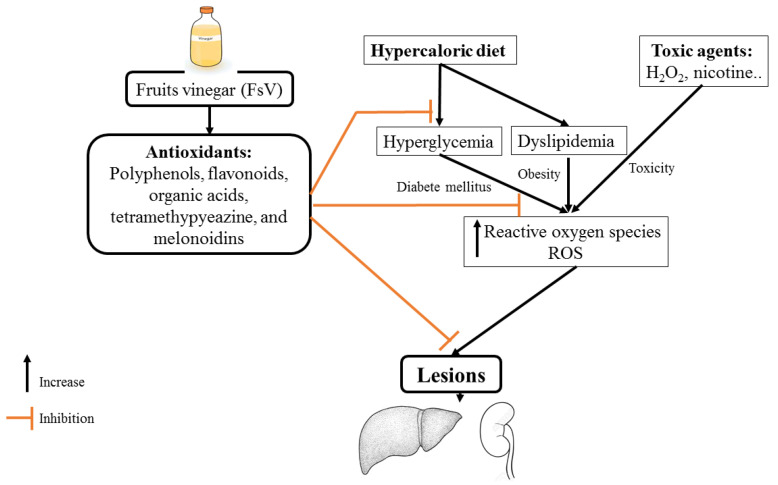
Mechanism of action possible of apple vinegar on nephro-hepatic functions against hydrogen peroxide and d-glucose toxicities.

Investigations of the antihyperglycemic activity of FsV were started in the late 80s. In fact, the research conducted by [87] has demonstrated that the co-administration of 2% acetic acid with meals (starch intake) decreased significantly glycemia. Subsequently, multiple studies also found that the administration of apple vinegar reduced blood glucose levels. This effect may be due in part to the stimulation of glucose uptake and enhancement of the action of insulin in skeletal muscle [63]. These beneficial effects have been attributed to acetic acid which acts via MAPK pathway (Figure 1). It is activated through a cascade of phosphorylation/dephosphorylation of proteins which induces inhibition of gene expression [18]. Subsequently, reduction of glucose-6-phosphatase, reduction of Phosphoenolpyruvate carboxykinase (PEPCK), and reduction of Sterol Regulatory Element-Binding Protein-1 (SERBP-1) which decrease blood glucose levels, stimulation of glucose storage, amelioration of insulin sensitivity, and decreases insulinoresistance (Figure 1) [18]. Recently, the administration of apple vinegar at a dose of 2 mL daily by gavage for five weeks decreases the risk of developing persistent hyperglycemia induced by a hypercaloric diet [16]. It was previously reported that the organic acids counteract hydrolyzing enzymes activities such as sucrase, trehalase, maltase, and lactase [88,89]. 

#### 5.1.2. Human Studies 

Research conducted on humans was initiated early in ancient Greece medicine, vinegar is prescribed as a remedy for several ailments [30]. Healthy subjects who consumed vinegar, which contains 1 g of acetic acid, combined with meals enriched with carbohydrates limited glycemic response due to its acidity without affecting gastric emptying [90]. Furthermore, vinegar consumption reduced the area under the insulin response curve by 20% after ingestion of sucrose, and the glycemic response was reduced by 30% [89]. Additionally, the ability of vinegar to stimulate glucose uptake by the forearm muscles and blood flow rates were investigated using the arteriovenous difference technique. Authors reported that the administration of vinegar decreases postprandial glycemia and improves the insulin action on skeletal muscle, which enhances glucose disposal [63]. Studies focused on the safety and tolerance of vinegar intake reported that vinegar consumption could induce bowel movements and flatulence. On other hand, Johnston et al. reported the hypoglycemic incidence on one of the patients treated with vinegar [22,58].

### 5.2. Antihyperlipidemic Effect

Vinegar is used in traditional medicine to treat dyslipidemia, which promotes the development of cardiovascular diseases [91]. The administration of apple vinegar during 8 weeks ameliorates lipid profile (cholesterol, low-density lipoprotein (LDL), and triglycerides) [91]. Additionally, mice fed with a hyper-fat diet and treated with synthetic acetic acid vinegar or nipa vinegar reduced total cholesterol, triglycerides, LDL, and leptin levels [92]. In another study, the treatment of 3T3-L1 cells with tomato vinegar decreased the triglycerides content by 45.71% as compared to cells untreated with tomato vinegar. It suppressed lipid accumulation in 3T3-L1 cells and inhibited adipogenic differentiation, which involves reducing fat accumulation mass and hepatic steatosis [93]. The molecular mechanism involved in the control of body fat by pomegranate vinegar was evoked for the first time by [94] who proved that the pomegranate vinegar activated AMPK in adipose tissue. The activation of AMPK inhibits anabolic pathways through suppression of SERBP-1c (Figure 1) [18,19,94]. Acetic acid is the main component present in vinegar. It has been shown that the acetic acid downregulates the expression of lipogenic genes through the activation of AMPK, subsequently inducing diminution of fatty acid synthase and Acetyl-CoA carboxylase levels [95,96]. The interaction of the phytochemical content of vinegar could be an excellent AMPK activator that may control lipids metabolism (Figure 1). 

### 5.3. Antimicrobial Effect

**Figure 2:**
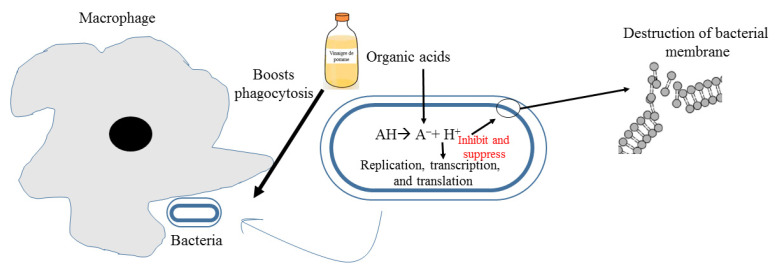
Mechanism of action possible of apple vinegar against pathogenic bacteria.

Bacterial resistance poses major health threats around the world on inconceivable scales. Vinegar has been shown to have potent antibacterial activity against resistant bacteria [28,85,97,98,99]. Recently, Yagnik et al. studied the effect of apple vinegar on the multiplication of two resistant bacterial strains (methicillin-resistant *Staphylococcus aureus* and *Escherichia coli* resistant to cefepime and cefepime-enmetazobactam combined). Proteomic analysis of both bacteria after treatment with apple vinegar shows the absence of key enzymes for DNA replication, glycolytic and respiratory proteins. Proteins absent after apple vinegar treatment for *E. coli* are 30 s ribosomal proteins, DNA-directed RNA polymerase alpha subunit, elongation factor TU and G OS-*E. coli*, formate C-acetyltransferase 1 OS *E-coli*, chaperone protein, 60 kDa chaperone OS *E. coli*. Concerning *S. aureus*, proteins not detected after treatment with apple vinegar are elongation factor TU and phosphoglycerate kinase [99]. Vinegar contains different organic acids that enter through bacterial membrane inciting internal pH decrease, protonation of macromolecules, and destabilization of the cell membrane by liberation of proton H^+^ (Figure 2) [100,101,102]. Other studies have shown a remarkable antibacterial potency of vinegar as compared with different detergents against several microorganisms [33,103]. On the other hand, antifungal activity of vinegar was established against *Candida albicans* spp. involved in dental stomatitis [104]. The combination of apple vinegar with the Endovac irrigation system shows promising results in irradiating *Enterococcus faecalis* (ATCC29212)[44]. 

### 5.4. Antioxidant Effect

The ability to counteract the deleterious effects of free radicals was the main property searched in a natural product. This property gives vinegar an important preventive and therapeutic effects. Various compounds were found in the vinegar viz polyphenols that have an interesting antioxidant potential due to their power to scavenge free radicals, chelate transition metal ions, and reduce oxidants (Figure 1) [7,8]. Furthermore, melanoidins are among the bioactive compounds in vinegar. Due to their negative charge and macromolecular properties, they have a strong ability to chelate transition metal ions preventing metal-induced oxidation reactions [105]. In addition, it contains a large system of conjugated π bonds and abundant reducing ketone structures, which gives it good free radical scavenging capacity and high reducing power [106]. Likewise, tetramethylpurazine has an antioxidant effect by stabilizing the levels of reactive oxygen species and increasing the levels of antioxidant enzymes including superoxide dismutase and catalase [43]. The antioxidant properties of fruits vinegar were previously studied. A panel of tests was used to determine the antioxidant ability of fruits vinegar. The study conducted by [25] revealed that the pomegranate vinegar exhibited high antioxidant activity as compared to Rioja wine vinegar, Young Sherry vinegar, Reserva Sherry vinegar, and Gran Reserva sherry vinegar. Additionally, the authors concluded that the antioxidant activity of pomegranate vinegar decreased during alcoholic fermentation by 17.6% during acetic fermentation. In a study developed with persimmon vinegar, apple vinegar, polished rice vinegar, and unpolished rice vinegar, researchers compared the antioxidant ability of vinegars and found that persimmon vinegar exhibited high superoxide radical-scavenging activity, DPPH radical-scavenging activity, hydroxyl radical-scavenging activity, and antioxidant activity against lipid peroxidation in tuna homogenates [107]. In vivo studies have shown that fruits vinegar increased enzymatic antioxidants including superoxide dismutase (SOD) (7 fold), glutathione peroxidase (GPX) (4.81 fold), glutathione reductase (GRx) (1.66 fold), and total antioxidant status (TAS) (3.45 fold). Additionally, fruits vinegar proved its ability to decrease TBARS levels both in serum and liver by 61.20% and 43.83% against hyper fat diet [32]. In the same line, Halima et al. [88] evoked that oral administration of fruit vinegar reduced malondialdehyde levels and increased enzymatic antioxidants in hyper fat-diet rats. Fruits vinegars can be useful for the attenuation of oxidative stress through enhancing enzymatic and non-enzymatic antioxidants levels. 

### 5.5. Anti-Inflammatory Effect 

Fruit vinegar had multiple uses as a healthy product. It is used against inflammation thanks to its ability to reduce the levels of inflammatory cytokines [39,108]. Previously, it proved that the pear vinegar ameliorates histological disorganization induced by dextran sodium sulfate (DSS) in an experimental animal model. Research experiments have proven that pear vinegar has an interesting ability in reducing serum IL-6 and IL-1ß concentrations [109]. Studies have been suggested vinegar as a potential liquid to overcome inflammation. In opposite, Ross and Poluhowich proposed that the administration of apple cider vinegar was ineffective against inflammation induced by injection of Freund’s complete adjuvant, which induces rheumatoid arthritis [110]. Recently, Beh et al. (2017) reported that the administration of Nipa vinegar inhibited the genes expression of nuclear factor-kappa B (NF-Kb) and inducible nitric oxide synthase (iNOS) in the liver of mice fed high-fat-diet, subsequently decreasing nitric oxide (NO) synthesis in live [92]. The consumption of apple vinegar inhibits cyclooxygenase-2, which suppresses proinflammatory markers expression and leads to diminish cytokines levels [111]. An experiment in vitro revealed that the co-culture of 3T3-L1 and Raw264.7 cells using the contact method with *Cudrania tricuspidata* fruits vinegar suppressed inflammatory markers expression decreasing nitric oxide (NO), inducible nitric oxide synthase (iNOS), tumor necrosis alpha (TNF-α), interleukin-6 (IL-6), monocyte chemoattractant protein-1 (MCP-1) [112]. 

### 5.6. Other Effects

Fruits vinegar contains diverse and different amounts of bioactive compounds which make it an active product against several diseases. Mounting evidence proved that fermented food counteracts several diseases including cancer [113]. Fruits vinegar contains also microbial components such as Lipopolysaccharides (LPS) that are generated during vinegar aging by the destruction of microorganisms by an elevation of acidity [114]. LPS modulates the host macrophage network to regulate numerous disorders such as diabetes, dyslipidemia, allergy, and cancer [115]. Additionally, it was proven that vinegar enhances phagocytic activity to ingest resistant microbes such as *Staphylococcus aureus* and *Escherichia coli,* which could reinforce the immune system to eradicate pathogen microbes and control the inflammation process [99]. Mimura et al. [116] investigated whether sugar can vinegar exerts an induction effect of apoptosis in human leukemia cells including HL-60, THP-1, Molt-4, U-937, Jurkat, Raji, and K-562. The finding led to the conclusion that sugar can vinegar contains lipophilic components with the ability to induce apoptosis in leukemia human cells. Study conducted by Seki et al. [117] revealed that the incorporation of vinegar in mice diet at a dose of 0.5% during 72 days decreased significantly tumor sizes and prolonged the life spans of mice implemented with sarcoma 180 and colon 38 tumor cells. Oxidative stress plays an important role in the installation of cancer through modifications of DNA. Furthermore, fruits vinegar exerts a good antioxidant ability as described above that could explain its antioxidative effect thereby protecting cells against reactive oxygen species damages.

Fruits vinegar consumption furnishes tremendous beneficial properties. It has been proven that vinegar prevents angiotensin-converting enzymes and reduced blood pressure in spontaneously hypertensive rats [95,118]. The mechanism of action was studied by Na et al. [119], who evoked that the vinegar decreased blood pressure through down-regulating AT1R expression through the AMPK/PGC-1/PPARγ pathway in spontaneously hypertensive rats at a dose of 7 mL/kg during 8 weeks of treatment. The experiment revealed that the treatment with vinegar inhibits the expression of angiotensin II receptor type 1 (AT1R) and increases the expression of PPARγ coactivator 1 alpha and PPARγ, which explains the antihypertensive effect of fruits vinegar.

## 6. Conclusions 

Vinegar is a healthy and wealthy product with multiple functional properties including antidiabetic, antihyperlipidemic, antimicrobial, and anti-inflammatory effects. Different techniques have unraveled the presence of different bioactive compounds in vinegar which are related to the raw material used to produce vinegar. Thus, bioactive ingredients are present or newly generated during brewing process. The synergetic effect between different components of vinegar provides several properties as mentioned above. 

## Figures and Tables

**Table 1:** Range values of physicochemical characteristics of vinegar.

Vinegar	Source	pH	Conductivity (mS/cm)	Acidity Titrable (%)	Ethanol (°)	Reference
Apple vinegar	Morocco	3.18–3.83	2.11–2.90	0.24–5.6	-	[28]
Apple vinegar	Algeria	-	-	0.73 ± 0.06	-	[32]
Pomegranate vinegar		-	-	0.98 ± 0.01	-	
Prickly pear vinegar		-	-	0.31 ± 0.02	-	
Date vinegar	Iraq	2.85–3.07	1.81–7.48	2.85–7.24	0.01–1.44	[31]
Date vinegar	Syria	2.99–3.23	5.43–3.91	4.22–5.26	1.07–2.53	
Ginger vinegar		3.26	3.86	5.04	2.88	
Grape vinegar		2.95	2.98	4.63	0.50	
Garlic vinegar		3.12	4.26	4.98	0.01	
Grape vinegar	Lebanon	2.49–2.86	1.31–2.84	5.34–6.18	0.01–0.18	
Apple vinegar	Turkey	2.71	1.72	5.17	0.09	
Grape vinegar		2.99	3.45	5.11	0.01	
Vegetable vinegar	USA	2.40	1.57	6.12	0.16	
Grape vinegar		2.53	1.56	5.40	0.18	
Sugarcane vinegar	KSA	2.43	1.57	6.36	0.01	
Grape vinegar	Turkey	2.70–3.90	-	0.32–5.72	-	[29]
Apple vinegar		2.71–3.56	-	0.66–7.20	-	
Artichoke vinegar		3.79 ± 0.00	-	1.22 ± 0.03	-	
Pomegranate		2.88–3.69	-	1.04–3.38	-	
Apple-lemon		3.64 ± 0.01	-	1.36 ± 0.03	-	
Hawthorne vinegar		3.76 ± 0.02	-	0.82 ± 0.03	-	
Lemon vinegar		2.63 ± 0.02	-	4.34 ± 0.07	-	
Sour sherry vinegar		3.05 ± 0.01	-	5.50 ± 0.07	-	
*Eucommia* *ulmoides* leaves vinegar	China	-	-	1.6–4.7	-	[33]
Wood vinegars	Thailand	2.90–3.50	-	2.72–4.92	-	[34]
Blueberry vinegar	Brazil	2.94–2.98	-	4.2–4.8	0.1–0.2	[35]
Apple vinegar	Romania	-	-	3.9–9	-	[27]
Dates vinegar	Malaysia	2.70–2.77	-	1.19–5.86	-	[36]
Citrus vinegar	Italy	2.65–3.24	-	2.96–13.32	0	[37]

**Table 3:** Organic acids in FsV.

Vinegars	Country	Organic Acids *	References
Apple vinegar	China	Tartaric acid, malic acid, lactic acid, citric acid, succinic acid	[5]
Red wine vinegar		Tartaric acid, succinic acid	
White wine vinegar		Tartaric acid, succinic acid	
Balsamic vinegar		Tartaric acid, malic acid	
Apple vinegar	Spain	Propionic acid, isobutyric acid, butyric acid, isovaleric acid	[72]
baby corn silk vinegar	Thailand	Dimethyl ether, 2-Ethylthio-2-methoxy-3-oxo-n-phenylbutanamide, 1-Butanol, 3-methyl-, acetate, Cyclobutane,1,1,2,3,3-pentamethyl-Isoamylalcohol, pentanoic acid, Butanedioic acid, diethyl ester, 3-Cyclohexene-1-methanol, 4-rimethyl -ethyl 2-phenylacetate, 4-[1′-Phenylethenyloxymethyl] pyridine, hexanoic acid, 1H-Pyrrolizine-7-carboxylic acid, 2-(formyloxy), octaboic acid	[73]
Sour cherry vinegar	Turkey	Isobutyric acid, isovaleric acid, hexanoic acid, octanoic acid, nonanoic acid, decanoic acid, dodecanoic acid, tetradecanoic acid, methyl acetate, ethyl acetate, ethyl propanoate, isobutyl acetate, isoamyl acetate, ethyl caproate, ethyl caprylate, ethyl decanoate, benzyl acetate, phenethyl acetate, 2-ethyl acetate, 2-ethyl hexanoic acid, ethanol, isobutyl alcohol, hexanol, nonanol, benzyl alcohol, phenethyl alcohol, 1-dodecanol	[50]
Pineapple vinegar	Thailand	Methyl ester, ethyl acetate, isobutyl acetate, isobutanol, isopentyl alcohol (3-methyl butanol), acetoin, benzaldehyde, propanoic acid, butanoic acid, isobutyric acid, 4-methylbenzaldehyde, methylbutanoic acid, naphthalene, phenylethel acetate, phenylethyl alcohol	[74]

* Acetic acid is present in all FsV.

**Table 4:** Mineral composition of FsV (mg/L).

Vinegars	Country	K	Na	Ca	Zn	Mg	Fe	P	Ni	Cr	Co	Mn	Reference
Banana vinegar	Romania	-	14.69–186.06	11.89–148.94	0.569–3.61	7.12–113.31	-	-	0.07–0.15	0.04–3.63	0.01–0.02	0.09–4.01	[82]
Apple vinegar	Turkey	802.24 ± 114	360.21 ± 250.380	104.75 ± 28.695	ND	65.60 ± 7.565	1.31 ± 0.585	48.06 ± 17.044	0.01 ± 0.013	-	-	0.18 ± 0.130	[79]
Rice vinegar		0.43 ± 0.056	ND	ND	ND	ND	0.62 ± 0.103	0.22 ± 0.020	0.01 ± 0.003	-	-	0.22 ± 0.011	
Sour cherry vinegar		1058.93 ± 103.502	303.20 ± 38.562	670.80 ± 30.811	0.32 ± 0.013	142.60 ± 46.11	10.68 ± 0.591	63.14 ± 11.078	0.12 ± 0.008	-	-	0.67 ± 0.009	
Date vinegar		1384.93 ± 132.745	181.40 ± 25.787	1136 ± 105.112	0.02 ± 0.024	195.60 ± 22.235	10.44 ± 2.526	70.32 ± 36.123	0.16 ± 0.008	-	-	0.28 ± 0.018	
Balsamic vinegar		1557.73 ± 416.841	264.96 ± 26.766	188.28 ± 46.997	0.36 ± 0.162	127.04 ± 18.470	6.94 ± 1.498	182.60 ± 50.577	0.03 ± 0.019	-	-	1.31 ± 0.180	
Apple vinegar	Morocco	32.403–41.863	0.039–0.199	1.569–2.620	0.014–4.212	1.572–1.746	0.499–0.581	-	-	-	-	0.045–0.053	[78]
Date vinegar	Algeria	0.14–2.73	23.6–30.9	0.24–0.79	-	0.16–1.92	0.22–1.74	-	-	-	-	-	[83]
Date vinegar	Iraq	1958	148	293	1.29	50	1.15	-	-	-	0.069	0.49	[31]
Wood vinegar	China	7.66 ± 0.80	-	13 ± 0.78	0.166 ± 0.16	1.98 ± 0.34	3751 ± 60	-	-	-	-	23.7 ± 0.43	[80]

## Data Availability

Data are available upon request.

## Sample Availability

Samples of the compounds are not available from the authors.
